# Multiple Molecular Mechanisms to Overcome Multidrug Resistance in Cancer by Natural Secondary Metabolites

**DOI:** 10.3389/fphar.2021.658513

**Published:** 2021-05-21

**Authors:** Mahmoud Zaki El-Readi, Ahmed M. Al-Abd, Mohammad A. Althubiti, Riyad A. Almaimani, Hiba Saeed Al-Amoodi, Mohamed Lotfy Ashour, Michael Wink, Safaa Yehia Eid

**Affiliations:** ^1^ Department of Biochemistry, Faculty of Medicine, Umm Al-Qura University, Makkah, Saudi Arabia; ^2^ Department of Biochemistry, Faculty of Pharmacy, Al-Azhar University, Assiut, Egypt; ^3^ Department of Pharmaceutical Sciences, College of Pharmacy & Thumbay Research Institute for Precision Medicine, Gulf Medical University, Ajman, United Arab Emirates; ^4^ Pharmacology Department, Medical Division, National Research Centre, Cairo, Egypt; ^5^ Department of Pharmaceutical Sciences, Pharmacy Program, Batterjee Medical College, Jeddah, Saudi Arabia; ^6^ Department of Pharmacognosy, Faculty of Pharmacy, Ain Shams University, Cairo, Egypt; ^7^ Institute of Pharmacy and Molecular Biotechnology, Heidelberg University, Heidelberg, Germany

**Keywords:** multidrug resistance, apoptosis, secondary metaabolites, cancer, molecular mechanism

## Abstract

Plant secondary metabolites (SMs) common natural occurrences and the significantly lower toxicities of many SM have led to the approaching development and use of these compounds as effective pharmaceutical agents; especially in cancer therapy. A combination of two or three of plant secondary metabolites together or of one SM with specific anticancer drugs, may synergistically decrease the doses needed, widen the chemotherapeutic window, mediate more effective cell growth inhibition, and avoid the side effects of high drug concentrations. In mixtures they can exert additive or even synergistic activities. Many SM can effectively increase the sensitivity of cancer cells to chemotherapy. In phytotherapy, secondary metabolites (SM) of medicinal plants can interact with single or multiple targets. The multi-molecular mechanisms of plant secondary metabolites to overcome multidrug resistance (MDR) are highlighted in this review. These mechanisms include interaction with membrane proteins such as P-glycoprotein (P-gp/MDR1); an ATP-binding cassette (ABC) transporter, nucleic acids (DNA, RNA), and induction of apoptosis. P-gp plays an important role in the development of MDR in cancer cells and is involved in potential chemotherapy failure. Therefore, the ingestion of dietary supplements, food or beverages containing secondary metabolites e.g., polyphenols or terpenoids may alter the bioavailability, therapeutic efficacy and safety of the drugs that are P-gp substrates.

## Plant Secondary Metabolites Biosynthesis Pathways and Classification

All higher plants have the capacity to produce secondary metabolites (SM) Table 1.

**TABLE 1:** Estimated number of plant secondary metabolites (Wink, 2010).

Type of secondary metabolite	Estimated numbers
*Nitrogen-containing*	
Alkaloids	21,000
Non-protein amino acids (NPAAs)	700
Amines	100
Cyanogenic glycosides	60
Glucosinolates	100
Alkamides	150
Lectins, peptides, polypeptides	2,000
*Without nitrogen*	
Monoterpenes (C10)	2,500
Sesquiterpenes C15)	5,000
Diterpenes (C20)	2,500
Triterpenes, steroids, saponins	5,000
Tetraterpenes (C40)	500
Flavonoids, tannins	5,000
Phenylpropanoids, lignin, coumarins, lignans	2,000
Polyacetylenes, fatty acids, waxes	1,500
Polyketides	750
Carbohydrates	200

**FIGURE 1:**
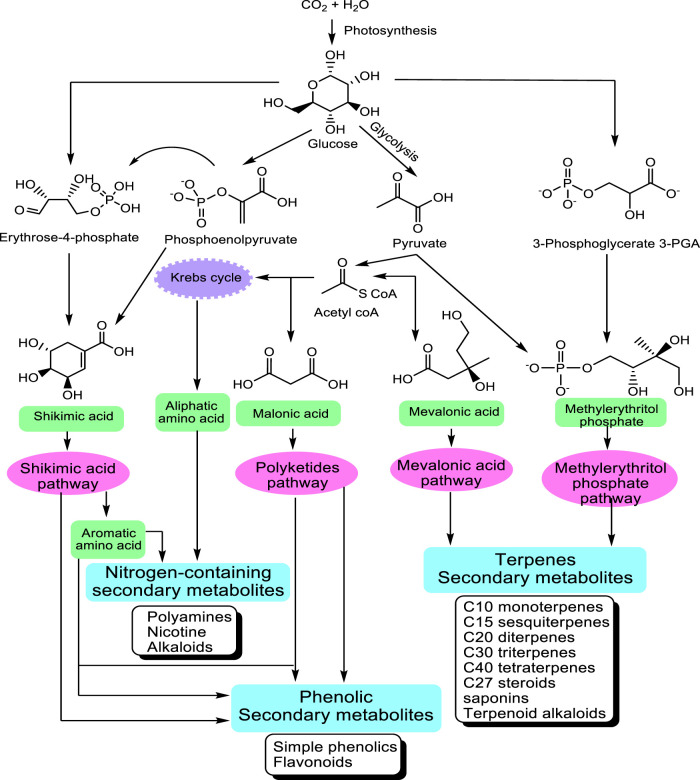
Main pathways leading to SM modified from Wink (2010). The non-mevalonate pathway or 2-C-methyl-D-erythritol 4-phosphate/1-deoxy-D-xylulose 5-phosphate pathway (MEP/DXP pathway) of isoprenoid biosynthesis is an alternative metabolic pathway leading to the formation of isopentenyl pyrophosphate (IPP) and dimethylallyl pyrophosphate (DMAPP) and finally terpenes.

The great majority of these metabolites are derived from five precursor pathways, acetyl coenzymeA (polyketides such as anthraquinones, flavonoids), active isoprene (various terpenenoids), shikimic acid (aromatic amino acids, cinnamic acids, tannins, indole and isoquinoline alkaloids), glycolysis (sugars, gallic acid), and tricarboxylic acid cycle; krebs cycle (alkaloids) (Figure 1). At present, more than 140,000 structurally diverse SM have been produced and identified from these pathways (Wink, 2010).

The structurally diverse SM can be classified based on the presence or absence of a nitrogen group in their structures into two large groups: SM with nitrogen in their structures and those without nitrogen. In Table 1, an estimate of the numbers of known SM is given in the table above.

### Biosynthesis Site and Storage of Plant Secondary Metabolites

**FIGURE 2:**
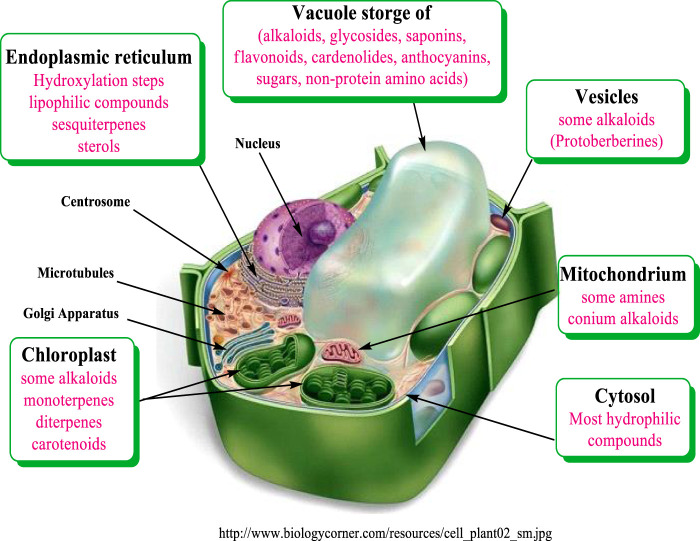
Compartments of biosynthesis and storage for plant secondary metabolites modified from Wink (1999).

Most biosynthetic pathways completely, or at least partially, occur in the cytoplasm, in the endoplasmic reticulum or in organelles. Hydrophilic compounds are usually stored in the vacuole. The lipophilic substances are sequestered in laticifers, resin ducts, glandular hairs, or on the cuticle. Many terpenoids (such as monoterpenes, diterpenes, and carotenoids) are synthesized through the pyruvate/glyceraldehyde phosphate pathway, or in the chloroplast (Wink, 1999). The corresponding genes are apparently localized in the cell nucleus. Sesquiterpenes are formed mainly in the endoplasmic reticulum (ER) Figure 2.

### Function of Secondary Metabolites for the Plants

SMs have vital functions, which are important for the fitness of their plants. Their main roles include a defense function against herbivores (insects, vertebrates), fungi, bacteria, viruses, and other plants. Some SM act as signal compounds to attract fertilizing and seed-dispersing animals. Others serve as communication-signals between the plants and symbiotic microorganisms (mycorrhizal fungi or N-fixing Rhizobia). Moreover, SMs have a protection role against UV light or other physical stress (Wink, 2003).

Our study focuses on a selection of SM which represents structures from each chemical class, such as glaucine, harmine, and sancguinarine for alkaloids and menthol, aromadendrene, β-sitosterol, β-carotene, crocin, retinoic acid, canthaxanthin, fucoxanthin, and digitonin (saponin) for terpenes and epigallcatechinegallate (EGCG) and thymol for polyphenols. In order to understand the role of this set of representative SM in cancer treatment, it is important to introduce notes about the cellular targets, mode of action and mechanisms of anticancer activity of SM classes.

### Major Cellular Targets for Secondary Metabolites

**FIGURE 3:**
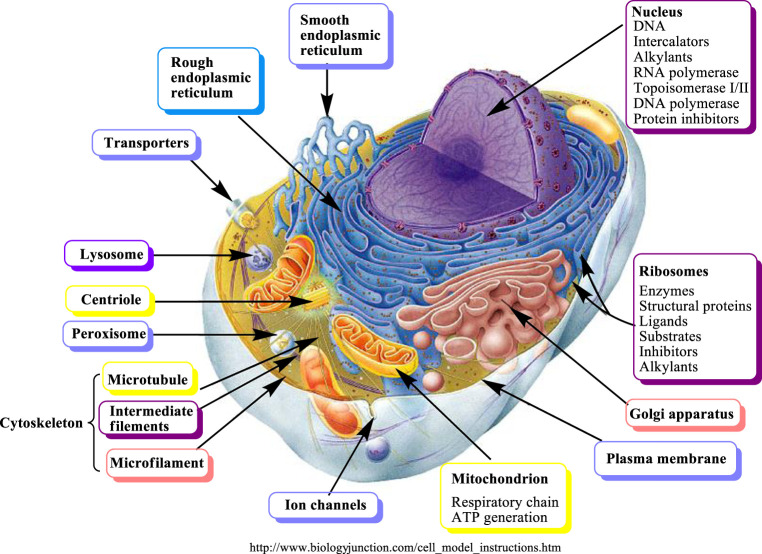
Molecular targets of secondary metabolites modified from (Wink, 2007).

SMs interfere with several important molecular targets present in a cell (Figure 3).

SM can be classified into two major groups according to their biological activities (Wink, 2008):

(1) SM which are highly selective for cellular targets and developing strong biological activity.
(2) SM which are non-selective and show moderate or weak bioactivity. They can attack various cellular targets. This non-selective type includes broad spectrum phytochemicals, which may occur in multicomponent mixtures and can exert synergistic interactions.

There are four major cellular targets for SM including:

(1) Proteins are represented in different forms such as receptors, enzymes, ion channels, transporters, regulatory proteins(Alexander et al., 2019a; Alexander et al., 2019b; Alexander et al., 2019c; Alexander et al., 2019d; Alexander et al., 2019e; Alexander et al., 2019f; Alexander et al., 2019g), cytokines, chemokines (Khare et al., 2019), structural proteins, cytoskeletal proteins, microtubules forming the mitotic spindle, transcription factors, and hormones. MHC-B complex genes that encode for cell surface proteins, are activated and affect the tumor outcome (Khare et al., 2018). It has been reported that most SM apparently interact with proteins and change the protein conformation in an unselective manner (binding, complexing, and denaturing). SM with reactive functional groups, such as aldehyde, epoxide or terminal and/or exocyclic methylene, and cyclopropane groups (found in several terpenes), are able to undergo electrophilic or nucleophilic substitutions allowing the formation of covalent bonds with a protein by binding to free amino-, SH- or OH- groups. This covalent interaction might lead to changes in the 3D conformation and thus loss of protein activity; or it might alter the protein turnover due to the inability of proteases to break down the alkylated protein. Polyphenols are example of SM which change the three-dimensional structures of proteins (Wink, 2008; Wink, 2010; Wink and Schimmer, 2010).
(2) Bio-membrane (fluidity, permeability). Saponins and other lipophilic terpenoids are examples of SM which influence the bio-membrane. Saponins are the glycosides of triterpenes or steroids, including steroidal alkaloids and cardiac glycosides. The bidesmosidicsaponins (inactive) cleave into the monodesmosidicsaponins (active) by β-glucosidase. Monodesmosidicsaponins, such as digitonin, have an amphipathic nature, their lipophilic terpenoid moiety is able to make a complex with cholesterol in biomembranes, while their sugar side chain binds to surface glycoproteins and glycolipids (Wink and Van Wyk, 2008). As a result, transient or permanent pores are generated in the membrane and make it leaky. This unspecific activity affects a wide set of cells and can easily be demonstrated with erythrocytes, which lose their haemoglobin when in contact with monodesmosidicsaponins (Wink, 2008; Wink and Van Wyk, 2008; Wink and Schimmer, 2010).
(3) Nucleic acids (DNA, RNA). Alkylating or intercalating SM affect a wider range of animals and microorganisms. The ability of SM to affect more than one molecular target is a typical SM behavior; therefore, additive and even synergistic activities can be expected. Generally, the SM that target membranes and DNA have cytotoxic activity and the affected cells usually undergo apoptosis (Wink, 2000; Wink, 2007). In addition, the covalent modifications are one major cause of point-mutations (deletion or exchange of one or more nucleic acid base) as well as failure of repair enzymes to exchange the modified bases (Wink, 2000).
(4) Inhibition of energy generation or cell division can lead to cell death, especially apoptosis (Pratt et al., 2020).

SM exert several effects on cancer cells *in vitro*, and on tumors *in vivo* (experimental animals), as well as interacting with anti-cancer drugs (affecting positively or negatively their efficacy), and protecting normal host organism tissues from the adverse effects of anti-cancer therapies (Wink, 2015; Seca and Pinto, 2018). These effects can be summarized as the follows:

#### DNA Alkylation

All genes are carried in DNA which encodes all proteins that are important for metabolism and normal cell development. Thus DNA is a main target of many SM (Roberts and Wink, 1998). Alkylating agents target N7 guanine in DNA. These compounds add methyl (anion, cation, or free radical) or other alkyl groups and form covalent bonds with the N7 guanine of DNA strands lead to strand breaks. Alkylguanine-DNA alkyl transferase (AGT) is a repair enzyme, which able to remove this modification. If the repair processes are failed, several types of mutation are arising such as nucleotide exchanges (transitions and transversions) or deletions. This leads to the inhibition of the correct utilization of guanine by base pairing and causes a miscoding of DNA and frame-shift mutation, translated gene into nonsense protein, and consequently, loss of protein function (Wink, 2007). Alkaloids e.g. aristolochic acids and pyrrolizidine alkaloids, lactones e.g. 3-propiolactone and parasorbic acid, glycosides e.g. cycasin and macrozamin are examples of classes of plant SM that exert alkylation of DNA (Wink, 2007).

#### DNA Intercalation

DNA intercalation is a non-covalent interaction between small molecules (mutagenic agents) and DNA (Persil and Hud, 2007). The intercalation is a result of the hydrophobic interactions between appropriate functional groups of SM and stacked base pairs of DNA. It is followed by ionic attraction, as expected between several cationic SM, such as alkaloids and nucleic acid (anionic). Intercalating SM is generally characterized as aromatic, planar, and hydrophobic. These features of SM lead to the hypothesis that they could very easily form π–π complexes with other planar aromatic molecules, such as nucleotide bases in DNA; adenine-thymine (AT) and guanine-cytosine (GC) (Wink and Schimmer, 2010; Hill et al., 2011). This interaction stabilizes the double-stranded DNA; therefore, DNA replication or transcription is stopped (Schmeller et al., 1997; Roberts and Wink, 1998).The intercalation also takes place in RNA double-stranded stem structures due to complementary base pairing (Wink, 2007). Frame-shift mutation is considered as a trigger of some lethal effects of intercalation on cells, which leads to changes in the sequence of corresponding amino acid (Wink, 2007). There is a positive correlation with the strongest being the intercalation and the inhibition of DNA and RNA processing enzymes, such as DNA polymerase I and reverse transcriptase (RT) (Roberts and Wink, 1998). Examples of intercalating agents are sanguinarine, harmine, glaucine, berberine, camptothecin, chelerythrine, doxorubicin, caffeine, theophylline, acridine orange, and ellipticine (Bathaie et al., 2007; Wink and Van Wyk, 2008; Hill et al., 2011). It has been reported that intercalating and alkylating SM induce apoptosis or inhibit carbohydrate-processing enzymes. Swainsonine**,** a Golgi a-mannosidase II inhibitor, is an example of alkaloids that intercalate with many glycosyltransferase enzymes leading to change in the produced carbohydrates, which participate in cell-cell and cell-substratum interactions affecting processes such as lymphocyte trafficking, immune cell stimulation, embryogenesis, and cancer metastasis (Goss et al., 1995).

#### DNA Topoisomerase

DNA replication, repair and recombination are essential processes for living cells. Topoisomerases (topo) are located in the nucleus of the cell; they alter the supercoiling and prevent entanglement of DNA double strands during DNA replication, transcription, exchanges of DNA segments between chromosomes (recombination) and elimination of erroneous DNA sequences (repair) (Wang et al., 2014). Therefore, topoisomerase inhibitors lead to cell death by blocking these essential processes. Mammalian cells contain two types of topoisomerases (topo) I and II. Topo I acts by transiently cutting single strands of DNA whereas topo II break double strands of DNA. Both types of topoisomerase break DNA via a catalytic tyrosine attack on a phosphodiester bond on the DNA backbone. The inhibition of topoisomerase occurs either directly by inhibiting this catalytic activity or by stabilizing the cleavable ternary complexes (CTC) by preventing strand resealing and converting CTC to lethal lesions that result when the cell tries to use the damaged DNA template for replication (Bailly, 2000; Ganguly et al., 2007; Wink, 2007; Alberts, 2008). Inhibition of one type of topoisomerase is enough to lead to cell-cycle arrest and cell death by apoptosis while synergistic cytotoxicity is observed when inhibiting both types of topoisomerase (Wink, 2007). Topoisomerase I is an important target of several SM in cancer cell; it can be targeted by several alkaloids such as quinazoline–quinoline, camptothecin, quinolones, and also by several flavones (Alison, 2002; Ma et al., 2004). A number of SM affect topo I and II enzymes such as sanguinarine, indenoquinolone, coralyne, acridine, and celastrol (triterpene) (Gatto et al., 1996; Makhey et al., 1996; Lin et al., 2005; Wink, 2007).

#### Telomeres and Telomerase

Human telomeres are tandem repeats of the hexameric sequence TTAGGG at the ends of linear chromosomes. The telomere sequence has a role in maintaining chromosomal integrity through its ability to prevent degradation, recombination, and be mistaken for DNA double-strand breaks (Shay and Wright, 2005). After finishing the replication many times, the telomeres are lost in subsequent cell divisions, leading to replicative senescence (Levy et al., 1992). Telomerase is a ribonucleoprotein enzyme responsible for the *de novo* synthesis of telomeres by adding telomeric DNA onto 3’ ends of chromosomes (Greider and Blackburn, 1985). Telomerase is an interesting target for anticancer therapy because it is necessary for the growth and survival of cancer cells. SM can trigger a DNA damage response, either via the “drug stacking” model of interaction with telomeres, or by inhibiting telomerase enzymatic activity. Inhibitor combinations (with different mechanism of actions) could accelerate telomere shortening and reduce the period of time required for killing cancer cells (Hathcock et al., 2004; Alberts, 2008). The combination of a major SM of green tea (EGCG and retinoic acid) inhibits telomerase activity (Yokoyama et al., 2008). Alkaloids including tetrandrine, fangchinoline, berbamine, ellipticine, cryptolepine, and neocryptolepine, terpenoids including genistein, ginsenoside, and ginseng saponin are examples of telomerase inhibitors from natural plant SM (Alberti et al., 2003; Guittat et al., 2003; Garbett and Graves, 2004; Liu et al., 2004; Ouchi et al., 2005; Zhang et al., 2005; Wang and Fang, 2006; Wink, 2007; Ji et al., 2011).

#### Cytoskeleton

The cytoskeleton (CSK) is structural protein classified into microfilaments (actin filaments), intermediate filaments, and microtubules. Microtubules play an important role in maintaining cell structure, providing platforms for intracellular transport, and assembling the spindle during mitosis (cell division); as well as, other cellular processes, e.g. apoptosis (Gierke et al., 2010). Microtubules are responsible for completing the mitosis process of cell division by separating the duplicated chromatids and pulling each part into daughter cells. Tubulin α- and β- dimers are polymerized into proto-filaments. A group of 13 proto-filaments laterally associate to form a long hollow cylindrical structure of microtubules. GTP molecules promote the polymerization (assembly) and bind it with both α- and β-tubulin dimer (Alberts, 2008). GTP-α-tubulin complex is stable while GTP-β-tubulin complex is unstable and it might be hydrolyzed to GDP soon after assembly. Therefore GDP-tubulin is prone to depolymerization and fall off (disassembly) (Wink, 2007). The dynamic instability of microtubules leads to cell cycle arrest (at mitosis) and subsequently apoptosis (Zhou and Giannakakou, 2005). The inhibition of the microtubule assembly or disassembly becomes a major target for anticancer agents because the cell division in cancer cells is faster than differentiated normal cells (Wink, 2007). It has been reported that almost all natural SM which have anticancer activity can interact with microtubules (Wink, 2007). Vinblastine and vincristine inhibit microtubule assembly while Taxol^®^ (diterpene alkaloid) inhibits the microtubule disassembly and blocks cell division in the late G2 phase (Lobert et al., 1996; Wink, 2007). Chelidonine, colchicine, sanguinarine, phalloidin, and noscapine are examples of SM that interacting with microtubules (Wink, 2007).

#### Induction of Apoptosis

Apoptosis, or programmed cell death, is an intrinsic death process that plays an important role during development and in adult life (Kerr et al., 1972). Therefore, too little apoptosis or escape from apoptosis can promote carcinogenesis and progression (Bellamy et al., 1995). In addition, the inability of cancer cells to undergo apoptosis after treatment is one of the main reasons for the development MDR in cancer cells (Gimenez-Bonafe et al., 2009). Thus, induction of apoptosis is considered to be a novel approach to overcoming MDR in cancer treatment. The apoptosis pathways are the receptor pathway or extrinsic pathway that involve stimulation of the tumor necrosis factor (TNF) superfamily e.g. TNF-related apoptosis induces ligand (TRAIL) receptors or CD95 (APO-1/Fas) and the mitochondrial pathway (intrinsic). In the extrinsic pathway, the stimulation of death receptors by natural SM ligands or other stimuli, initiate the oligomerization of receptors followed by the recruitment of adaptor molecules (e.g. FADD procapase-8) to the stimulated death receptors, which leads to the induction of caspase-8 and finally an increase of caspase-3 (Sayers, 2011). Alternatively, caspases-8 may cleave Bid (BH3 protein of the Bcl-2 family) into tBid which activates the outer membrane permeabilization of mitochondria. Therefore, Bid is considered a link between the extrinsic and the intrinsic pathways (Walczak and Krammer, 2000). In the intrinsic pathway DNA damage and p53 stimulate the release of inter-membrane space proteins into the cytosol. These proteins include cytochrome c and/or second mitochondria-derived activators of caspase (Smac)/direct IAP binding protein with low pI (DIABLO), which trigger the activation of effector caspases (Tait and Green, 2010). Cytochrome c is involved in the apoptosome complex formation (cytochrome c + Apaf-1 + caspase-9) which leads to subsequent activation of the caspase-9, then induction of caspase-3 (Alberts, 2008; Tait and Green, 2010). However, Smac/DIABLO is able to bind and antagonize the inhibitor of apoptosis proteins (IAP) including survivin, XIAP, c-IAP2, c-IAP1, and livin/melanoma-IAP (ML-IAP) promoting the activation of caspases-3, -7 and -9 (LaCasse et al., 2008). There are several proteins that can control apoptosis pathways. Pro-apoptotic proteins include multi-domain proteins (e.g. Bak and Bax) and BH3 domain-proteins (e.g. Bid, Noxa, Bim, and Puma) and anti-apoptotic proteins such as the Bcl-2 family (e.g. Bcl-2, Mcl-1, and Bcl-xL), which significantly participate in intrinsic pathway regulation (Tait and Green, 2010). Examples of SM that induce apoptosis are sanguinarine, harmine, colchicine, vinblastine, berberine, emetine, and cinchonine(Rosenkranz and Wink, 2008; Cao et al., 2011; Chan, 2011; Hamsa and Kuttan, 2011; Lee et al., 2012).

#### Interaction With ABC Transporters

**FIGURE 4:**
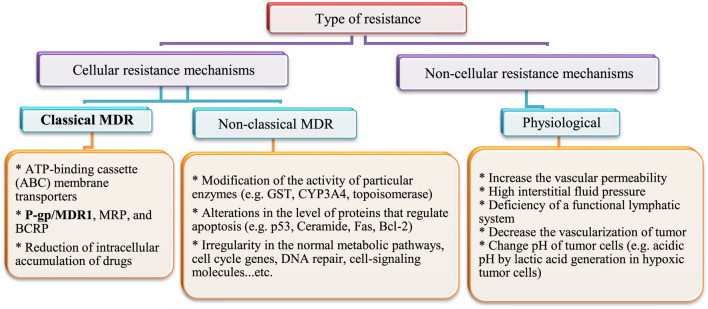
Mechanisms of multidrug resistance (MDR) in cancer cells.

After heart diseases, cancer is the second-leading cause of death worldwide. Annually; more than 19.3 × 10^6^ cancer cases and 10 × 10^6^ cancer deaths are reported (Ferlay et al., 2010). The rate of cancer death is expected to increase worldwide, with an estimated 10 × 10^6^ humans dying from cancer in 2020 and 28.4 × 10^6^ dying in 2040 (Cho, 2007; Jemal et al., 2011). In addition, cancer is a multi-factorial disease resulting in abnormal and unlimited division of cells. Treatment strategies that inhibit growth of cancer cells cause some serious side effects on normal cells and cancer cells often develop resistance to drug treatment. Drug resistance develops not only to single drugs, but can also occur to several classes of drugs, even those with diverse chemical structures and modes of action. This is known as multidrug resistance (MDR). This might explain why treatment with cytotoxic agents or even multiple agent combinations with different targets is sometimes not effective. Several types of drug resistance have been identified (Fan et al., 1994). They have been classified into cellular and non-cellular mechanisms, as summarized in Figure 4.

This section will highlight the role of membrane transporters in the classical type of multi-drug resistance.

##### Membrane Transporters

Membrane transporters are classified into two main families: The ATP binding cassette (ABC) transporter family and the solute carrier (SLC) transporter family (Choudhuri and Klaassen, 2006).

###### The ATP-Binding Cassette (ABC) Transporters

**FIGURE 5:**
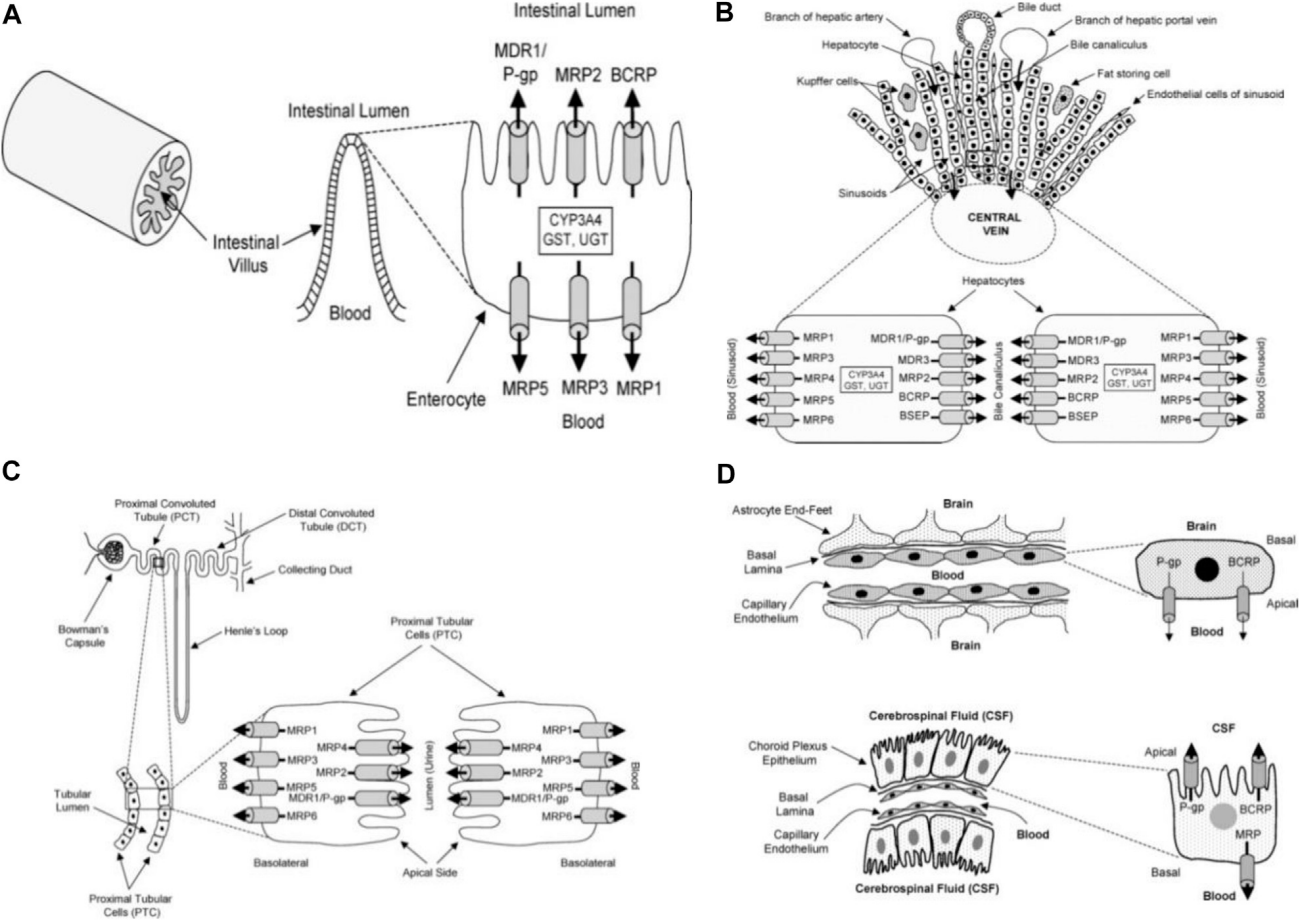
Diagrammatic section of the small intestine **(A)**, liver **(B)**, proximal tubular cells of kidney **(C)**, choroid plexus epithelium and blood brain barrier **(D)** adapted from Choudhuri and Klaassen (2006).

Several substrates (lipids, bile acids, xenobiotic, and peptide antigens) are transported against a concentration gradient by ATP-binding cassette (ABC) transporters proteins. This active process is powered by ATP hydrolysis. ABC-transporters are expressed in different organs such as the small and large intestine, kidney, adrenal gland, placenta, liver, and capillary endothelial cells of brain and testis (Cordon-Cardo et al., 1989; Fricker et al., 1998; Fricker, 2008).The tissue localization of ABC-transporters suggests that they play a physiological function in the detoxification process. They transport and reduce the amount of exogenous and endogenous substances in the body and excrete the xenobiotics and metabolites into the gastrointestinal lumen, bile, and urine (Figure 5). Thus, ABC-transporters play an important role in the absorption and distribution of drugs; consequently, determining the pharmacokinetic properties and clinical response of several drugs (Gutmann et al., 1999; Lin and Yamazaki, 2003; Fricker and Miller, 2004; Mahringer et al., 2011). However, drug resistance can develop through this action when therapeutic agents are also effluxed from cells and eliminated from the body.

##### Classification of (ABC) Transporters

Christopher Higgins coined the term “ABC transporter” in 1992 (Higgins, 1992). Today, 49 human ABC transporter family genes have been identified. They are classified into 7 subfamilies (number of members): ABCA (12), ABCB (11), ABCC (13), ABCD (4), ABCE (1), ABCF (3), and ABCG (5). There are three subfamilies which confer important drug resistance and they are expressed in several organs; ABCB1 (MDR1/P-glycoprotein of subfamily ABCB), subfamily ABCC (MRPs), and ABCG2 (BCRP of subfamily ABCG). Italics and numerical suffixes are used to designate genes e.g. *ABCB/MDR* (italicized) with a numerical suffix, such as *ABCB1* (or *MDR1*), *ABCC1* (or *MRP1*); whereas, a lack of italics indicates a protein as ABCB1 (or MDR1), ABCC1 (or MRP1) (Efferth, 2001; Gottesman et al., 2002).

###### P-glycoprotein (P-gp)

P-gp or MDR1 is a polypeptide dimer (1280-residue), which represents a pore forming membrane protein. It was the first studied member of ABC transporters. P-gp has long been of interest to molecular and cellular biologists because its overexpression is linked to MDR in human cancers. It transports many types of drugs such as cytotoxic agents, immuno-suppressants, protease inhibitors, statins, calcium channel blockers, steroids, beta-blockers, anticonvulsants, antihistamines, and antidepressants (Takara et al., 2006). Differing stimuli, including light ultraviolet (UV), retinoic acid, phorbol esters, butyrate, and several chemotherapeutics can induce P-gp gene expression. Additionally, environmental and/or genetic factors likely influence P-gp expression (Greiner et al., 1999).

###### Multidrug Resistance Protein (MRP)

**FIGURE 6:**
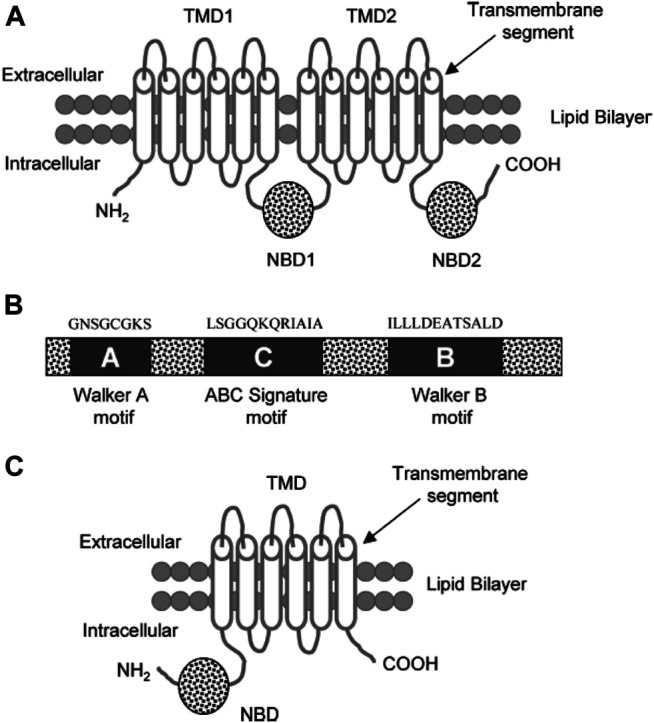
**(A)** Diagram of a typical ABC transporter protein inserted in the lipid bilayer. The trans-membrane protein consists of 2 trans-membrane domains (TMDs) and the two nucleotide ATP binding domains (NBDs) **(B)** The NBDs consists of walker A, walker B, and ABC signature C motifs with characteristic residues of the respective amino acid sequences above these motifs. **(C)** ABCG2 diagram showing a half transporter (6 trans-membrane segments and 1 NBDs) at the N-terminal end (Choudhuri and Klaassen, 2006).

It was originally believed that the overexpression of P-gp (170-kDa) alone led to multidrug resistance in cancer cells. Eight additional MRPs that potentially contribute to drug resistance have since been discovered. The multidrug resistance protein 1 (190-kDa; MRP1) is located in most organs in the basolateral membrane of epithelial cells (Fricker and Miller, 2002) (Figure 6). It appears to play an important role in protecting the cells from bilirubin toxicity. MRP1 is able to transport many anticancer agents, such asanthracycline antibiotics (e.g., doxorubicin, daunorubicin), epipodophyllotoxins (e.g. etoposide, teniposide), and *Vinca* alkaloids (e.g. vincristine, vinblastine) (Gottesman et al., 2002).

###### Breast Cancer Resistance Protein (BCRP)

ABCG2 (also called BCRP/ABCP/MXR) is an ABC transporter that is about half the size of P gp (∼70-kDa). It contains six trans-membrane segments and one NBD at the N-terminal end in the cytoplasmic side. It is an efflux transporter found in most tissues and is clinically relevant as another mediator of drug resistance (Kühnle et al., 2009).

##### General Structural of ABC Transporter (Mainly P-gp)

A typical ABC transport protein consists of two parts sharing a high level of sequence uniformity. One half is composed of two hydrophobic trans-membrane domains (TMDs) and the other of two hydrophilic nucleotide-binding domains (NBDs) found at the cytoplasmic face of the membrane. Each of the NBDs is associated with one of the TMDs; NBD1 with TMD1 and NBD2 with TMD2 (Figure 6). There are usually six trans-membrane segments in each TMD; therefore, 12 segments (*α*-helices) in total are located in the apical part of the cell membrane (Raviv et al., 1990; Rosenberg et al., 1997; Loo and Clarke, 1999).

Two of the TMDs of P-gp that are coupled with one another by a ∼75–amino acid linker region fold in a unique way (as known from Computer-assisted algorithms) (Rao and Nuti, 2003).The NBDs contain three characteristic motifs (Walker A, Walker B, and an ABC signature (C) motif). The ABC signature motif of NBDs is located across from the Walker A sequence of NBD2 in a sandwich configuration (Figure 6). Thus, ATPs are sandwiched between adjacent Walker A and ABC signature motifs (Akhtar et al., 2011). The three-dimensional structure with high resolution (25- Å) shows that there is a large central chamber in P-gp which is closed at the cytoplasmic side and opens to the lipid phase. This structure allows substrates to have free access from the cytoplasm, while denying access to substrates from the outer leaflet. A cross-sectional view of a P-gp molecule (4.5 Å) shows an overall clover-leaf shape and the central cavity is opened in the TMDs by conformational changes in the protein. These changes mediate substrate transportation (Rosenberg et al., 1997).

##### The Models and Mechanisms of P-gp Drug Efflux

###### The Classical Model

Simply, this model suggests that, P-gp actively expels substrate drugs from the cytoplasm to the extracellular locationthrough the two TMDs in a pore-forming arrangement (Borst and Schinkel, 1997).

###### Flippase Model

**FIGURE 7:**
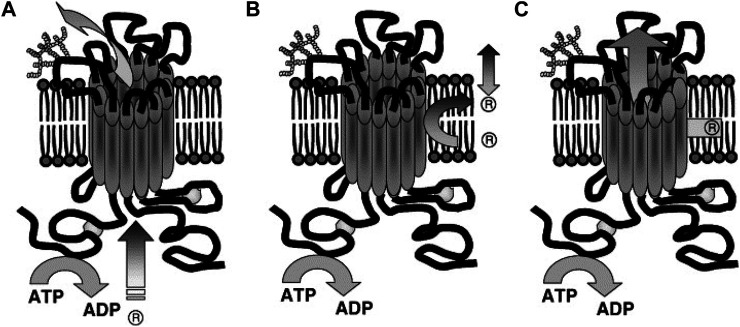
Suggested Models illustrating P-gp mechanisms of drug efflux. **(A)** Classical or pore, **(B)** flippase and **(C)** hydrophobic vacuum cleaner models. In the classical model: substrate effluxes out of the cell through a protein channel by interacting with P-gp in the cytoplasm. In the flippase model: substrate transports into the inner leaflet, binds to P-gp (within the membrane), translocates to the outer leaflet, and passively diffuses into the extracellular. In the hydrophobic vacuum cleaner model: the characteristics of ‘pore’ and ‘flippase’ models are mixed (Li et al., 2007).

The substrate-binding site of P-gp is located on the inner face of the plasma membrane and in front of the cytoplasmic face. The hydrophobic portions of the substrate molecule are oriented toward the hydrophobic core of the membrane whereas the charged portion is oriented toward the polar cytosolic face of the membrane (Figure 7) (Homolya et al., 1993).

The transport stages in this model are 1) the substrate is laterally diffused until it binds to the substrate-binding site in the inner leaflet of the lipid bilayer (pocket in the cytoplasmic leaflet). 2) The ATPase activity of P-gp hydrolyzes ATP and allows the protein to flip the substrate into the outer leaflet. 3) Then the substrate diffuses outside of the cell into the extracellular aqueous phase or else the outer leaflet (Higgins and Gottesman, 1992). It is believed the drug release from P-gp includes re-orientation of the drug binding site from the inner leaflet (cytosolic side) to the outer leaflet (extracellular side) of the membrane and is followed by a change from high to low drug binding affinity. Thus, it is hypothesized that ATP binding, rather than hydrolysis, is the driver behind the conformational changes which accompany the transport process (Rosenberg et al., 2001; Rosenberg et al., 2003). This flipping triggers the rearrangement of the P-gp structure, expelling the substrate. For example, in the lipopolysaccharide (sugar head and lipid tail) molecule as a substrate model, the hydrophilic heads are sequestered and then “flipped” in the internal chamber dragging the lipophilic tails through the bilayer as suggested by its “flipping” structure (Regev et al., 2005).

###### Hydrophobic Vacuum Cleaner Model

P-gp recognizes substrates as foreign to the cell membrane and pumps them from the intracellular compartment into the extracellular medium or from the lipid bilayer, thereby detecting and eliminating xenobiotic and hydrophobic drugs as they penetrate the lipid bilayer, much like a hydrophobic vacuum cleaner (Gottesman and Pastan, 1993). The vacuum cleaner model suggests that the lipid bilayer is integral to drug concentration, whereas P-gp itself has a low intrinsic substrate binding affinity (Sharom, 2014). This model suggests that substrates and modulators partition into membranes prior to interacting with the transporter (Figure 7C). P-gp substrates typically contain high lipid/water partition coefficients. They are concentrated at very high levels within the membrane (Regev et al., 2005).

##### P-Glycoprotein Substrates

P-gp can interact with hundreds of substrates, which are diverse in chemical structure and action, such as those found in natural products, anti-cancer drugs, cardiac glycosides, calcium channel blockers, analgesics, immunosuppressive agents, anthelminthic, HIV protease inhibitors (HPI), antibiotics, H2-receptor antagonists, fluorescent dyes, steroids, and peptides (linear and cyclic) (Amin, 2013).

Generally, P-gp substrates come in a variety of sizes, ranging from small (e.g. daunorubicin and doxorubicin) to large complex molecules (e.g. paclitaxel and vinblastine). Researchers have not yet fully defined the structural activity relationship of P-gp substrates; however, it is known that most substrates are hydrophobic organic cations, amphipathic, having a wide range of molecular weight (300‒2000 Da), and contain a minimum of two planar rings (Seelig, 1998; Cianchetta et al., 2005). Substrates may be anionic, cationic, or uncharged (Ueda et al., 1997). Structural characters required for the compound to interact with P-gp are made up of either two or three of electron donors (hydrogen bond acceptors) with a fixed spatial separation configuration (Seelig, 1998; Cianchetta et al., 2005). It seems to be a direct correlation between the affinity of a compound for P-gp and its number of hydrogen bonds plus its lipophilicity (Ecker et al., 1999). The three dimensions quantitative structure activity relationship (3DQSAR) study shows that the interaction between the substrate with the binding sites of P-gp is crucial to efflux (Xing et al., 2009).

TMDs of P-gp have a high number of amino acids in the side chains which are able to interact with substrates and act as hydrogen bond donors (Seelig, 1998; Cianchetta et al., 2005). In addition, the aromatic amino acid residues of substrates may have a role in the binding and transporting of these substrates (Pawagi et al., 1994). Therefore, the efficiency of a substrate to bind is dependent on how many points are simultaneously interacting with P-gp which in fact depends on the chemical structure of the drug. Van der Waals and hydrophobic interactions have a role in substrate binding because of the electrostatic interactions between charged amino acid in the side chains of P-gp and charged functional groups on the drug molecules (Watkins et al., 2001). Furthermore, a high concentration of the substrate in the membrane is important for enhancing the affinity interaction of the substrate with P-gp (Cascorbi, 2011). Additionally, the lipid environment surrounding P-gp can modulate the ATPase activity of P-gp and affect coupling of the drug binding site with NBDs.

##### P-Glycoprotein Modulators

Additionally, another class of compounds "modulators" interacts with P-gp. These are also known by other names such as MDR reversal agents or chemosensitizers. Modulators have similar structural properties to substrates which interact in the drug binding pocket and are transported by P-gp. Generally, substrates with a high affinity and high trans-bilayer diffusion rate are effective modulators. Effluxed compounds can re-enter the outer leaflet and then diffuse across the membrane (or "flip-flop" into the inner leaflet), where they once again interact with P-gp and are re-exported. Substrates re-enter the membrane slowly enough that the P-gp can keep up, establishing a drug gradient, and developing drug resistance (Li-Blatter et al., 2012). On the other hand, modulators re-enter so quickly that the P-gp cannot efflux them fast enough, causing a futile cycle to operate such as Rho123 which has a t_1/2_ of 3 min (Eytan et al., 1996). The performance of modulators has been shown to be related to their diffusion rate beyond the membrane (Eytan and Kuchel, 1999). Transporter turnover and ATP hydrolysis rates are high while no concentration gradient is generated, resulting in cells that are not resistant to modulators. MDR modulators can inhibit the activity of the P-gp efflux pump. Therefore, they have the ability to potentiate cytotoxicity, making them valuable alternatives for use in overcoming MDR (Robert and Jarry, 2003). Even though, cells are not resistant to modulators, they can be effectively affected by substrates combined with modulators (Eckford and Sharom, 2009; Sharom, 2011). Secondary metabolites (SM) of plants including phenolics, terpenoids, and alkaloids are intriguing candidate for use in chemosensitizing or reversing MDR in ABC transporters expressed in cancer cells (Wink et al., 2012). Most terpenes (mono-, di-, tri-, and sesquiterpenes) are very hydrophobic SM which can bind to bio-membranes. At higher doses, these SM are able to unselectively disturb membrane fluidity and permeability causing uncontrolled efflux of metabolites and ions and possibly even cell leakage. Terpenes can stimulate death receptors, induce apoptosis, and modulate proteins in the membrane (Wink and Van Wyk, 2008).The aforementioned modulators interfere with P-gp's ability to extrude drugs and exert a drug concentration gradient; thus, reversing MDR in cancer cells *in vitro*. It is clinically important because it can act as a selective blocker of the P-gp efflux pump and improve drug uptake and bioavailability (Fu and Arias, 2012).

##### Established P-Glycoprotein Inhibitors/Modulators

Some drug substrates of P-gp can inhibit P-gp mediated efflux or uptake of other P-gp substrates. Several P-gp inhibitors can inhibit P-gp function either by binding to drug-binding sites of the membrane transport proteins as competitive inhibitors or via indirect mechanisms by stopping the ATP hydrolysis process (non-competitive inhibitors). Indirect mechanisms are related to phosphorylation of the transport proteins or the expression of the P-gp gene (Sikic, 1999; Wink et al., 2012). Ca^2+^ channel blockers (e.g. verapamil, diltiazem, nifedipine, nicardipine, quinidine, quinine), antimicrobial agents (e.g., ketoconazole, itraconazole, erythromycin, cefoperazone, ceftriaxone, clarithromycin), HPIs (ritonavir, indinavir, saquinavir, nelfinavir), benzimidazole proton pump inhibitors (PPIs) or gastric H^+^/K^+^-ATPase inhibitors (e.g. omeprazole, lansoprazole, pantoprazole, rabeprazole), tamoxifen, propranolol, hydrocortisone, and progesterone have been shown to inhibit P-gp activity, leading to potentially relevant drug interactions (Pauli-Magnus et al., 2001; DuBuske, 2005; Fricker, 2008).

Additionally, several herbal constituents which are often used by cancer patients; as well as, dietary phytochemicals, are used as complementary and alternative medicines (CAMs). They can modulate or inhibit P-gp activity and/or expression (Fricker, 2008; Mahringer et al., 2010; Efferth and Greten, 2012; Eichhorn and Efferth, 2012). Recently, the effect of many natural occurring polyphenols, terpenoids, and alkaloids on P-gp expression and activity were reviewed (Wink et al., 2012). In resistant cancer cells, P-gp, MRP1, and BCRP can be competitively inhibited by some alkaloids (isoquinoline, protoberberine, quinoline, indole, monoterpeneindole, and steroidal alkaloids), lipophilic terpenoids (mono-, di-, tri-terpenes including saponins), tetraterpenes (including carotenoids) (Eid et al., 2020), and steroids (including cardiac glycosides) (Wink et al., 2012). Remarkably, initial observations also show that many more polar phenolic SM (phenolic acids, flavonoids, xanthones, chalcones, catechins, anthocyanins, tannins, stilbenes, anthraquinones, and naphthoquinones) (El-Readi et al., 2019) can directly disrupt the 3D structure of P-gp, thus inhibiting its activity in MDR cancer cells (Wink, 2008). There is a need for specific studies with ABC- transporters (mainly P-gp) with the end goal of gaining knowledge which leads to the development of new drugs and/or therapies to improve human quality of life. The next section will focus on the combination of plant secondary metabolites as an approach to overcoming MDR and exerting synergistic interactions.

### Combination Therapy to Overcome MDR

Drug combinations have been used for the treatment of a wide range of serious diseases including cancer, HIV, AIDS, TB, malaria, diabetes, hypertension, MRSA etc. Drug combination has a long history in herbal medicines such as traditional Chinese medicines (TCM) which is considered as poly-pharmacy with many multidrug regimens (Efferth, 2010).

Today, drug combinations are more established and developed because of the valuable advances in the technology of phytochemical isolation science and chemical synthetic capability. Over 90% of cancer deaths are related to MDR, and thus a failure in chemotherapy (Coley, 2010).

Finding a way to overcome MDR in cancer cells is the main focus of chemotherapy research which is mainly concerned with a combinational strategy. The use of many agents with different mechanisms of action may target several targets or diseases. The probable outcome advantages of drug combination include:

(1) Enhancing the efficacy of the therapy.
(2) Reducing the dose while enhancing or maintaining the same efficacy to prevent toxicity.
(3) Overcoming or reversing MDR (El-Readi et al., 2019; Abdalla et al., 2020; Abdallah et al., 2020).
(4) Achieving selective synergism against a target vs. host (Chou, 2006).

The effect of the drug combinations can be classified into three types: synergistic; the biological effects of the drugs in combinations are greater than the sum of individual drug effects, additive; the effects of the drug combinations are equal to additional effects and antagonistic; the biological activity of drugs in combination are lower than the sum of the individual drug effects. Efficient strategies as seen in “theoretical basis experimental design and computerized simulation in combinational drug studies” have been reported by Chou (2006). The combinational index, isobologram, and the correlation coefficient of linear regression depending on the medium effect equation are the most proper methods to apply when evaluating the drug combination.

#### Definition of Synergy

There are many definitions for the term “synergy”. The “isobole method” is considered as one of the most common and demonstrative methods to prove a proposed synergistic effect (Berenbaum, 1989). In an isobologram of isobole methods, the *x* and *y* axes reflect the dose of the single drugs A and B that exert certain effects (e.g. IC_50_) and the various doses in combinations are evaluated for the same effect. An isobole is represented by a line or curve between points of the same effect. According to the Berenbaumisobole method, the interaction results can be classified into 3 categories; additive, antagonism, and synergism (Berenbaum, 1989). The additive effect or zero (0) interaction means that the effect of two drugs (A and B) is a pure summation effect of both individual drugs as the follows:

$$E\left(D_{A},D_{B}\right)=E\left(D_{A}\right)+E\left(D_{B}\right)$$

The universal observed effect (E); D_A_ and D_B_ are the doses of drugs A and B.

Antagonistic interaction means the combination effect is less than a predictable effect from the summation of the individual drug effects and the data points of a combination will create a convex curve.

$$E\left(D_{A},D_{B}\right)<E\left(D_{A}\right)+E\left(D_{B}\right)$$

In a synergistic interaction, the overall combination effect of two drugs (A and B) is greater than would be expected by the summation of the individual drug effects. The result of the combination data points is then a concave curve.

$$E\left(D_{A},D_{B}\right)>E\left(D_{A}\right)+E\left(D_{B}\right)$$

In synergism, lower doses of drugs A and B are required to achieve the desired effect. The synergistic effect can be quantified as fold or factors as doubling or even greater multiplication of the expected effect. The advantage of dose reduction of combined drugs is a probable decrease in the side effects of potent cytotoxic drugs.

#### Mechanisms of Synergy Effects

The mechanisms of synergistic interactions are usually unknown. However, it is important to gain knowledge regarding the mechanism of action of combined drugs before managing drug combination studies. For example combining P-gp-substrate drugs (vinblastine, paclitaxel, and doxorubicin) with P-gp-reversing agents (e.g. ningalin, ardeemin, and limonin) (Chou, 2006; El-Readi et al., 2010). The synergistic mechanisms can be classified into at least 4 different possible mechanisms depending on the pharmacological, biochemical, molecular biological and clinical effects (Wink, 2008; Wagner, 2011):

1. Synergistic multi-target effects: Plant SM targets not only one site, but many targets and can simultaneously interact in a synergistic fashion. In addition, the SM can act as a synergistic partner or different molecules of the same signalling cascade (multi-target effects (Wink et al., 2012). A good example of such synergetic effect is seen in case of the activity of *Urtica dioica* roots towards prostate hyperplasia, which has been associated with a synergism of antiproliferative and anti-inflammatory effects by lectins and polysaccharides (Efferth and Koch, 2011).
2. Pharmacokinetic and physiochemical effects: Plant SM can improve solubility and/or the resorption rate and thereby the bioavailability of the synergistic partner. For example, enhancing the solubility or absorption rate of the active SM via inactive by-products (e.g. tannins). Furthermore, it can be supposed that bioavailability, interaction with ABC- transporter pathways, deactivation of active SM to inactive SM or activation of inactive pro-drugs, prevention of binding to target proteins (e.g. by tannins)....etc. may take place as a part of the mode of action in the synergistic process (Wink, 2008; Efferth and Koch, 2011).
3. Reverse the resistance mechanisms of cancer cells (against chemotherapy). SM can increase intracellular accumulation of pharmacologically active compounds by inhibiting the P-gp efflux pump. For example, the interaction of 5-methoxy-hydnocarpine (P-gp inhibitor) and berberine (P-gp substrate) in *Berberis* plants (Stermitz et al., 2000).
4. Neutralization or elimination of the toxic effects of SM via combination with synthetic drugs which that improve the overall efficacy results. Additionally, some SM may induce phase I metabolizing enzymes, e.g. CYP3A4, which are responsible for changing the pro-drugs (those with no pharmacological activity) into active metabolites (El-Readi et al., 2013; Eid et al., 2015).

### Plant Secondary Metabolites and Combination Therapy

Plant secondary metabolites have important roles in chemotherapy (Eid et al., 2012a; Eid et al., 2012b; Eid et al., 2013). Over 60% of currently used chemotherapeutical agents are derived from natural sources such as plants, microorganisms, and marine organisms (Newman et al., 2003). More than 3350 plants species have been used in cancer treatment (Hartwell, 1982; Graham et al., 2000). However, most isolated plant compounds do not serve as drugs; rather, they provide lead structures for the development of potential novel agents. There are many examples of effective anticancer drugs or chemotherapeutical agents derived from plants, which are used for the treatment of many types of tumours including camptothecin, etoposide, epipodophyllotoxin such as paclitaxel (taxol), and *Vinca*alkolloids (vinblastine, vincristine) (Wink, 2007; Efferth, 2010). Ulrich-Merzenich et al., have mentioned several reasons for the interest in research into the development of a new generation of phytopharmaceuticals from natural sources by synergistic drug combinations (Ulrich-Merzenich et al., 2010).

Generally, hydrophobic SM, such as terpeneoids (including saponins), steroids (including cardiacglycosides) can modulate P-gp in cancer cells. Being lipophilic, they usually inhibit the active transporters by competing for binding to the active side, which present an excellent opportunity when administered as a chemosensitizer in combination with a cytotoxic agent. Limonin isolated from many Citrus species at 20 μM dose significantly enhanced doxorubicin cytotoxicity 2.98-fold and 2.2-fold in Caco2 and CEM/ADR5000 cells, respectively (El-Readi et al., 2010).

On the other hand, the alkaloids isolated from Chelidonium majus such as berberine chelidonine, and sanguinarine represent good examples for SMs that can synergistically act with many chemopreventive and epigenetic modifiers (HDACs inhibitors), which are widely used in cancer treatment. Concomitant treatment of chelidonine at 20 μM dose with doxorubicin dramatically decreased the IC50 of the known cytotoxic drug on Caco-2 cells to almost one tenth of the original (El-Readi et al., 2013). The other alkaloidal analogue sanguinarine reduced the IC_50_ value of doxorubicin in two-drug combinations (sanguinarine + doxorubicin) to 17.58-fold in the highly resistant Caco-2 cells (Eid et al., 2012; Eid et al., 2013). On the same context, berberine can also acts synergistically with vorinostat in SW480 colon cancer cells (Li et al., 2021).

Traditional systems of medicine, such as Chinese medicine, provide a lot of clinical prescriptions for many known diseases. The term “Reverse pharmacology” has been coined for traditional medicines and the biodiversity of medicinal plants has so far remained mainly unexplored and is still a valuable strategy for the finding of new drugs (Pieters and Vlietinck, 2005; Patwardhan and Mashelkar, 2009). However, the isolation and chemical identification of pure single SM has only resulted in moderate yield. For example, the National Cancer Institute, USA investigated the anticancer activity of 114,000 extracts (from about 35,000 plants) and only a few compounds from this large screening project have been clinically applied as chemotherapeutical agents such as paclitaxel and topotecan (Cragg and Boyd, 1996). Moreover, it is difficult to treat multifactorial diseases such as cancer, diabetes, or cardiovascular diseases with mono-target therapies. The synergy of multi-target therapies created by the combination of naturally-occurring phytochemicals is considered as a good approach to increase the efficacy of such treatment, and it might be cheaper than synthetic treatment (Vernon et al., 2010). In this study the pre-approval cost of new anticancer entities can be reduced to more than half of the total cost of two new anticancer agents in addition to the increased efficacy of such combinations. Hence, some combination therapies are currently in use in modern medical therapy for disease treatment. These are often used without documenting the original expected cumulative efficacy or adverse effects of both single treatments. Therefore, there is an increasing interest in the synergy concept (Ulrich-Merzenich et al., 2009).

In addition, the worldwide need for phytopharmaceuticals is steadily growing. The world-bank has reported that the trade in medicinal herbs, plant derived drug products, and crud plant materials is increasing annually by a growth rate between 5–15% (Patwardhan et al., 2005). For example, the herbal industry had a turnover of about 62 billion-dollars in 2005 (Patwardhan et al., 2005). Therefore, it is important to gain understanding about plant SM and their molecular and cellular targets to exert their cytotoxic effects on cancer cells.

## Conclusion

Many studies provide important information regarding the molecular targets of plants secondary metabolites. The drug-herbal and drug-diet interactions associated with ABC-transporters, mainly P-gp is very important topic in cancer therapy. Understanding drug efflux and uptake, knowing the effects of other phytochemicals and natural products on multidrug resistance mediated transport, and the mechanism of modulating the drug transport proteins is essential for predicting clinical interactions in humans. Further research could provide knowledge which might prevent potentially harmful drug-drug interactions due to altered chemotherapeutic efficacy, when plant SM contained in (for example), an herbal medicine, is taken concurrently with prescribed medicines.
